# Tris(3-hy­droxy­imino-1-methyl­indolin-2-one) monohydrate

**DOI:** 10.1107/S1600536811015418

**Published:** 2011-05-07

**Authors:** Yanqing Miao, Xiaoqing Zhang, Chunye Liu, Jing You

**Affiliations:** a Xi’an Medical University, Department of Pharmacy, Hanguang Road No. 137, Xi’an 710021, Xi’an, People’s Republic of China

## Abstract

There are three independent 3-hy­droxy­imino-1-methyl­indolin-2-one mol­ecules and a water mol­ecule in the asymmetric unit of the title compound, 3C_9_H_8_N_2_O_2_·H_2_O. The crystal packing is stablized by O—H⋯O and O—H⋯Nhydrogen bonds between 3-hy­droxy­imino-1-methyl­indolin-2-one mol­ecules and the water mol­ecule and weak π–π stacking inter­actions [centroid–centroid distances in the range 3.446 (2)–3.983 (2) Å], forming a two-dimensional network.

## Related literature

For the anti-bacterial, anti-virus and neuroprotective activity of indolin-2-one derivatives, see: Chen *et al.* (2009*a* ▶,*b* ▶, 2010*a* ▶,*b* ▶). For standard bond lengths, see Allen *et al.* (1987 ▶).
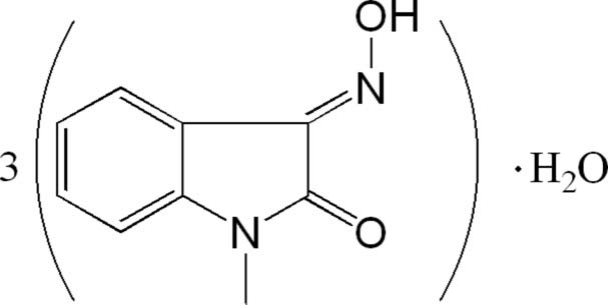


## Experimental

### 

#### Crystal data

3C_9_H_8_N_2_O_2_·H_2_O
*M* *_r_* = 546.54
Triclinic, 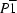

*a* = 8.920 (3) Å
*b* = 10.811 (4) Å
*c* = 14.915 (5) Å
α = 91.335 (3)°
β = 101.013 (3)°
γ = 112.784 (3)°
*V* = 1294.1 (7) Å^3^
*Z* = 2
Mo *K*α radiation
μ = 0.10 mm^−1^
*T* = 296 K
0.36 × 0.28 × 0.25 mm

#### Data collection

Bruker SMART CCD area-detector diffractometer
Absorption correction: multi-scan (*SADABS*; Sheldrick, 2005 ▶) *T*_min_ = 0.944, *T*_max_ = 0.986
7872 measured reflections
4512 independent reflections
3154 reflections with *I* > 2σ(*I*)
*R*_int_ = 0.024

#### Refinement

*R*[*F* ^2^ > 2σ(*F* ^2^)] = 0.047
*wR*(*F* ^2^) = 0.140
*S* = 1.05
4512 reflections
362 parameters
H-atom parameters constrained
Δρ_max_ = 0.26 e Å^−3^
Δρ_min_ = −0.21 e Å^−3^

### 

Data collection: *SMART* (Bruker, 2002 ▶); cell refinement: *SAINT* (Bruker, 2002 ▶); data reduction: *SAINT*; program(s) used to solve structure: *SHELXS97* (Sheldrick, 2008 ▶); program(s) used to refine structure: *SHELXL97* (Sheldrick, 2008 ▶); molecular graphics: *ORTEP-3* (Farrugia, 1997 ▶); software used to prepare material for publication: *WinGX* (Farrugia, 1999 ▶).

## Supplementary Material

Crystal structure: contains datablocks I, global. DOI: 10.1107/S1600536811015418/jj2082sup1.cif

Structure factors: contains datablocks I. DOI: 10.1107/S1600536811015418/jj2082Isup2.hkl

Supplementary material file. DOI: 10.1107/S1600536811015418/jj2082Isup3.cml

Additional supplementary materials:  crystallographic information; 3D view; checkCIF report

## Figures and Tables

**Table 1:** Hydrogen-bond geometry (Å, °)

*D*—H⋯*A*	*D*—H	H⋯*A*	*D*⋯*A*	*D*—H⋯*A*
O7*W*—H7*WA*⋯O6^i^	0.96	1.82	2.752 (3)	164
O1—H1*B*⋯N1^ii^	0.82	2.11	2.780 (3)	139
O7*W*—H7*WB*⋯O6^iii^	0.96	2.02	2.958 (3)	165
O7*W*—H7*WB*⋯N5^iii^	0.96	2.59	3.181 (3)	120
O3—H3*C*⋯O7*W*	0.82	1.78	2.579 (3)	165
O5—H5*C*⋯O4	0.82	2.05	2.753 (3)	144

Symmetry codes: (i) 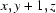; (ii) 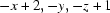; (iii) 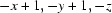.

**Fig. 1.**
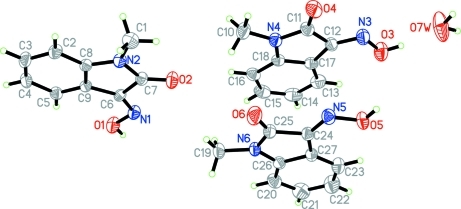
An ORTEP-3 drawing of the tile compound, with the atom-numbering scheme and 30% probability displacement ellipsoids.

**Fig. 2.**
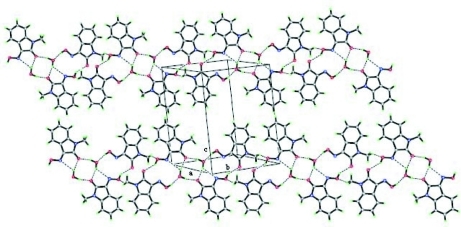
Packing of the title compound. Dashed lines indicate hydrogen bonds.

## supplementary crystallographic information

### Comment

Isatin derivatives attracted much attention related to their anti-bacterial, anti-virus and neuroprotection properties (Chen *et al.*, 2009*a*; Chen *et al.*, 2009*b*; Chen *et al.*, 2010*a*; Chen *et al.*,2010*b*). 3-(Hydroxyimino)-1-methylindolin-2-one, a related structure, has been synthesized by a condensation reaction of *N*-methyl isatin and hydroxylamine.  In this paper we report the X-ray crystal structure of the title compound, a related derivative of these bioactive compounds.

In the title compound, 3(C_9_H_8_N_2_O_2_), H_2_O, there are three independent 3-(hydroxyimino)-1-methylindolin-2-one planar molecules and a water molecule in the asymmetric unit (Fig. 1).  Bond lengthas and angles are in normal ranges (Allen *et al.*, 1087). Crystal packing is stablized by O—H···O hydrogen bonds between 3-(hydroxyimino)-1-methylindolin-2-one molecules and the water molecule and weak π—π stacking interactions (Table 2) forming a two-dimensional network (Fig. 2).

### Experimental

*N*-methyl isatin (1 mmol) was dissolved in methanol (20 ml), 10 ml me thanol solution of 1.2 mmol hydroxylamine was added dropwise, until the disappearance of isatin, as evidenced by thin-layer chromatography. The solvent was removed *in vacuo* and the residue was separated by column chromatography (silica gel, petroleum ether/ethyl acetate = 1:1), giving the title compound. 30 mg of the title compound was dissolved in 30 ml me thanol and the solution was kept at room temperature for 4 d, natural evaporation gave yellow single crystals suitable for X-ray analysis.

### Refinement

All H atoms were placed at calculated positions and refined as riding, with C—H = 0.93 Å, N—H = 0.86 Å and O—H = 0.82 Å, and with *U*_iso_(H) = 1.2*U*_eq_(C, N) or 1.5*U*_eq_(O).

### Crystal data

**Table:**

3C_9_H_8_N_2_O_2_·H_2_O	*Z* = 2
*M**_r_* = 546.54	*F*(000) = 572
Triclinic, *P*1	*D*_x_ = 1.403 Mg m^−^^3^
Hall symbol: -P 1	Mo *K*α radiation, λ = 0.71073 Å
*a* = 8.920 (3) Å	Cell parameters from 3022 reflections
*b* = 10.811 (4) Å	θ = 1.8–27.5°
*c* = 14.915 (5) Å	µ = 0.10 mm^−^^1^
α = 91.335 (3)°	*T* = 296 K
β = 101.013 (3)°	Block, yellow
γ = 112.784 (3)°	0.36 × 0.28 × 0.25 mm
*V* = 1294.1 (7) Å^3^	

### Data collection

**Table:**

Bruker SMART CCD area-detector diffractometer	4512 independent reflections
Radiation source: fine-focus sealed tube	3154 reflections with *I* > 2σ(*I*)
graphite	*R*_int_ = 0.024
φ and ω scans	θ_max_ = 25.1°, θ_min_ = 2.4°
Absorption correction: multi-scan (*SADABS*; Sheldrick, 2005)	*h* = −10→10
*T*_min_ = 0.944, *T*_max_ = 0.986	*k* = −12→12
7872 measured reflections	*l* = −17→16

### Refinement

**Table:**

Refinement on *F*^2^	Secondary atom site location: difference Fourier map
Least-squares matrix: full	Hydrogen site location: inferred from neighbouring sites
*R*[*F*^2^ > 2σ(*F*^2^)] = 0.047	H-atom parameters constrained
*wR*(*F*^2^) = 0.140	*w* = 1/[σ^2^(*F*_o_^2^) + (0.0716*P*)^2^ + 0.1735*P*] where *P* = (*F*_o_^2^ + 2*F*_c_^2^)/3
*S* = 1.05	(Δ/σ)_max_ < 0.001
4512 reflections	Δρ_max_ = 0.26 e Å^−^^3^
362 parameters	Δρ_min_ = −0.21 e Å^−^^3^
0 restraints	Extinction correction: *SHELXL97* (Sheldrick, 2008), Fc^*^=kFc[1+0.001xFc^2^λ^3^/sin(2θ)]^-1/4^
Primary atom site location: structure-invariant direct methods	Extinction coefficient: 0.029 (3)

### Special details

**Table:**

Geometry. All e.s.d.'s (except the e.s.d. in the dihedral angle between two l.s. planes) are estimated using the full covariance matrix. The cell e.s.d.'s are taken into account individually in the estimation of e.s.d.'s in distances, angles and torsion angles; correlations between e.s.d.'s in cell parameters are only used when they are defined by crystal symmetry. An approximate (isotropic) treatment of cell e.s.d.'s is used for estimating e.s.d.'s involving l.s. planes.
Refinement. Refinement of *F*^2^ against ALL reflections. The weighted *R*-factor *wR* and goodness of fit *S* are based on *F*^2^, conventional *R*-factors *R* are based on *F*, with *F* set to zero for negative *F*^2^. The threshold expression of *F*^2^ > σ(*F*^2^) is used only for calculating *R*-factors(gt) *etc*. and is not relevant to the choice of reflections for refinement. *R*-factors based on *F*^2^ are statistically about twice as large as those based on *F*, and *R*- factors based on ALL data will be even larger.

### Fractional atomic coordinates and isotropic or equivalent isotropic displacement parameters (Å^2^)

**Table:**

	*x*	*y*	*z*	*U*_iso_*/*U*_eq_	
C1	1.0271 (3)	−0.0706 (3)	0.09769 (15)	0.0623 (7)	
H1C	0.9774	−0.0060	0.0908	0.093*	
H1D	0.9527	−0.1539	0.0614	0.093*	
H1E	1.1302	−0.0366	0.0774	0.093*	
C2	1.1891 (3)	−0.2589 (2)	0.18264 (15)	0.0521 (6)	
H2A	1.1852	−0.2568	0.1200	0.063*	
C3	1.2550 (3)	−0.3391 (2)	0.23265 (16)	0.0574 (6)	
H3A	1.2959	−0.3919	0.2030	0.069*	
C4	1.2614 (3)	−0.3426 (2)	0.32544 (16)	0.0557 (6)	
H4A	1.3074	−0.3967	0.3576	0.067*	
C5	1.1999 (3)	−0.2663 (2)	0.37148 (15)	0.0478 (5)	
H5A	1.2032	−0.2694	0.4341	0.057*	
C6	1.0575 (2)	−0.0964 (2)	0.34600 (13)	0.0407 (5)	
C7	1.0105 (3)	−0.0384 (2)	0.25993 (14)	0.0448 (5)	
C8	1.1296 (2)	−0.18248 (19)	0.22847 (13)	0.0399 (5)	
C9	1.1338 (2)	−0.18576 (19)	0.32271 (13)	0.0386 (5)	
C10	0.8968 (3)	0.2792 (3)	0.09247 (17)	0.0675 (7)	
H10A	0.8865	0.2812	0.0273	0.101*	
H10B	0.8290	0.1906	0.1050	0.101*	
H10C	1.0111	0.3012	0.1215	0.101*	
C11	0.7880 (3)	0.4583 (2)	0.07539 (15)	0.0517 (6)	
C12	0.7441 (3)	0.5409 (2)	0.13851 (16)	0.0500 (6)	
C13	0.7619 (3)	0.5382 (2)	0.31557 (18)	0.0624 (7)	
H13A	0.7203	0.6035	0.3235	0.075*	
C14	0.8102 (3)	0.4762 (3)	0.38937 (18)	0.0704 (7)	
H14A	0.8017	0.5010	0.4477	0.084*	
C15	0.8700 (3)	0.3796 (3)	0.37811 (17)	0.0649 (7)	
H15A	0.9010	0.3399	0.4291	0.078*	
C16	0.8857 (3)	0.3390 (2)	0.29342 (15)	0.0536 (6)	
H16A	0.9263	0.2730	0.2861	0.064*	
C17	0.7775 (3)	0.5000 (2)	0.22938 (15)	0.0476 (5)	
C18	0.8386 (2)	0.4006 (2)	0.22021 (14)	0.0437 (5)	
C19	0.5818 (3)	0.0036 (2)	0.3080 (2)	0.0684 (7)	
H19A	0.6085	−0.0336	0.2572	0.103*	
H19B	0.5085	−0.0682	0.3355	0.103*	
H19C	0.6822	0.0540	0.3528	0.103*	
C20	0.4430 (3)	0.1584 (3)	0.42273 (17)	0.0636 (7)	
H20A	0.4850	0.1037	0.4570	0.076*	
C21	0.3780 (4)	0.2375 (3)	0.4624 (2)	0.0796 (8)	
H21A	0.3765	0.2360	0.5245	0.096*	
C22	0.3155 (4)	0.3182 (3)	0.4117 (2)	0.0831 (9)	
H22A	0.2712	0.3696	0.4399	0.100*	
C23	0.3178 (3)	0.3241 (2)	0.31945 (18)	0.0641 (7)	
H23A	0.2765	0.3796	0.2856	0.077*	
C24	0.4038 (3)	0.2266 (2)	0.18659 (15)	0.0457 (5)	
C25	0.4784 (3)	0.1244 (2)	0.18822 (17)	0.0513 (6)	
C26	0.4435 (3)	0.1635 (2)	0.33104 (15)	0.0469 (5)	
C27	0.3819 (3)	0.2464 (2)	0.27895 (15)	0.0459 (5)	
N1	1.0252 (2)	−0.05933 (18)	0.41960 (11)	0.0495 (5)	
N2	1.0588 (2)	−0.09364 (18)	0.19319 (11)	0.0463 (4)	
N3	0.6924 (2)	0.6279 (2)	0.10394 (15)	0.0642 (6)	
N4	0.8424 (2)	0.37665 (18)	0.12804 (12)	0.0487 (5)	
N5	0.3804 (2)	0.27722 (18)	0.11122 (13)	0.0546 (5)	
N6	0.5003 (2)	0.09227 (17)	0.27555 (13)	0.0506 (5)	
O1	1.0755 (2)	−0.11735 (17)	0.49500 (10)	0.0646 (5)	
H1B	1.0526	−0.0908	0.5404	0.097*	
O2	0.9441 (2)	0.04077 (16)	0.25021 (11)	0.0622 (5)	
O3	0.6579 (2)	0.69901 (18)	0.16874 (15)	0.0858 (6)	
H3C	0.6297	0.7564	0.1448	0.129*	
O4	0.7795 (2)	0.46187 (18)	−0.00736 (11)	0.0718 (5)	
O5	0.3164 (2)	0.37245 (16)	0.12083 (12)	0.0681 (5)	
H5C	0.3017	0.4030	0.0716	0.102*	
O6	0.5143 (2)	0.07832 (18)	0.12371 (13)	0.0762 (5)	
O7W	0.5777 (4)	0.8629 (2)	0.06685 (15)	0.1353 (11)	
H7WA	0.5613	0.9341	0.0977	0.162*	
H7WB	0.5520	0.8683	0.0019	0.162*	

### Atomic displacement parameters (Å^2^)

**Table:**

	*U*^11^	*U*^22^	*U*^33^	*U*^12^	*U*^13^	*U*^23^
C1	0.0753 (17)	0.0799 (17)	0.0359 (12)	0.0362 (15)	0.0094 (11)	0.0137 (12)
C2	0.0630 (15)	0.0597 (14)	0.0399 (12)	0.0292 (12)	0.0159 (11)	−0.0008 (10)
C3	0.0720 (16)	0.0551 (14)	0.0577 (15)	0.0365 (13)	0.0197 (12)	−0.0009 (11)
C4	0.0688 (16)	0.0486 (13)	0.0587 (15)	0.0329 (12)	0.0139 (12)	0.0087 (11)
C5	0.0580 (14)	0.0482 (12)	0.0416 (12)	0.0246 (11)	0.0131 (10)	0.0086 (10)
C6	0.0415 (12)	0.0465 (12)	0.0363 (11)	0.0193 (10)	0.0104 (9)	0.0000 (9)
C7	0.0463 (12)	0.0488 (12)	0.0417 (12)	0.0225 (11)	0.0074 (9)	0.0036 (10)
C8	0.0424 (12)	0.0422 (11)	0.0360 (11)	0.0177 (10)	0.0090 (9)	0.0020 (9)
C9	0.0412 (11)	0.0415 (11)	0.0334 (11)	0.0163 (10)	0.0091 (9)	0.0008 (9)
C10	0.0843 (18)	0.0856 (18)	0.0566 (15)	0.0555 (16)	0.0227 (13)	0.0133 (13)
C11	0.0531 (14)	0.0563 (14)	0.0443 (14)	0.0219 (12)	0.0067 (11)	0.0123 (11)
C12	0.0447 (13)	0.0442 (12)	0.0598 (15)	0.0175 (11)	0.0082 (11)	0.0106 (11)
C13	0.0627 (16)	0.0547 (15)	0.0646 (17)	0.0164 (13)	0.0189 (13)	−0.0066 (12)
C14	0.0768 (18)	0.0761 (18)	0.0458 (15)	0.0139 (15)	0.0215 (13)	−0.0022 (13)
C15	0.0686 (17)	0.0704 (17)	0.0432 (14)	0.0146 (14)	0.0108 (12)	0.0113 (12)
C16	0.0557 (14)	0.0573 (14)	0.0456 (13)	0.0199 (12)	0.0105 (11)	0.0137 (11)
C17	0.0433 (12)	0.0428 (12)	0.0503 (13)	0.0101 (10)	0.0106 (10)	0.0029 (10)
C18	0.0409 (12)	0.0470 (12)	0.0389 (12)	0.0133 (10)	0.0080 (9)	0.0050 (9)
C19	0.0577 (16)	0.0619 (16)	0.096 (2)	0.0363 (14)	0.0116 (14)	0.0238 (14)
C20	0.0660 (16)	0.0654 (16)	0.0571 (16)	0.0255 (14)	0.0082 (13)	0.0179 (13)
C21	0.095 (2)	0.087 (2)	0.0555 (16)	0.0337 (18)	0.0210 (15)	0.0055 (15)
C22	0.106 (2)	0.088 (2)	0.071 (2)	0.0529 (19)	0.0263 (17)	−0.0007 (16)
C23	0.0730 (17)	0.0614 (15)	0.0682 (17)	0.0383 (14)	0.0144 (13)	0.0039 (13)
C24	0.0420 (12)	0.0418 (12)	0.0513 (13)	0.0170 (10)	0.0047 (10)	0.0055 (10)
C25	0.0476 (13)	0.0466 (13)	0.0616 (15)	0.0211 (11)	0.0114 (11)	0.0063 (11)
C26	0.0390 (12)	0.0431 (12)	0.0544 (14)	0.0141 (10)	0.0052 (10)	0.0080 (10)
C27	0.0440 (12)	0.0419 (12)	0.0502 (13)	0.0177 (10)	0.0055 (10)	0.0041 (10)
N1	0.0602 (12)	0.0630 (12)	0.0351 (10)	0.0348 (10)	0.0112 (8)	0.0044 (8)
N2	0.0581 (11)	0.0580 (11)	0.0315 (9)	0.0319 (10)	0.0103 (8)	0.0076 (8)
N3	0.0594 (13)	0.0536 (12)	0.0829 (16)	0.0268 (11)	0.0128 (11)	0.0129 (11)
N4	0.0591 (12)	0.0560 (11)	0.0382 (10)	0.0301 (10)	0.0109 (8)	0.0094 (8)
N5	0.0518 (12)	0.0548 (12)	0.0553 (12)	0.0234 (10)	0.0024 (9)	0.0088 (9)
N6	0.0450 (11)	0.0459 (10)	0.0654 (13)	0.0240 (9)	0.0090 (9)	0.0129 (9)
O1	0.0917 (13)	0.0899 (12)	0.0370 (9)	0.0590 (11)	0.0210 (8)	0.0126 (8)
O2	0.0709 (11)	0.0703 (11)	0.0625 (11)	0.0475 (10)	0.0118 (8)	0.0094 (8)
O3	0.0981 (15)	0.0696 (12)	0.1084 (16)	0.0525 (12)	0.0243 (12)	0.0113 (11)
O4	0.0914 (13)	0.0919 (13)	0.0443 (10)	0.0493 (11)	0.0135 (9)	0.0213 (9)
O5	0.0724 (12)	0.0692 (11)	0.0749 (12)	0.0437 (10)	0.0087 (9)	0.0267 (9)
O6	0.0983 (14)	0.0796 (12)	0.0757 (12)	0.0561 (11)	0.0316 (11)	0.0080 (10)
O7W	0.260 (3)	0.135 (2)	0.0710 (14)	0.153 (2)	0.0142 (17)	−0.0009 (13)

### Geometric parameters (Å, °)

**Table:**

C1—N2	1.445 (3)	C15—C16	1.377 (3)
C1—H1C	0.9600	C15—H15A	0.9300
C1—H1D	0.9600	C16—C18	1.374 (3)
C1—H1E	0.9600	C16—H16A	0.9300
C2—C8	1.372 (3)	C17—C18	1.396 (3)
C2—C3	1.383 (3)	C18—N4	1.402 (3)
C2—H2A	0.9300	C19—N6	1.452 (3)
C3—C4	1.376 (3)	C19—H19A	0.9600
C3—H3A	0.9300	C19—H19B	0.9600
C4—C5	1.385 (3)	C19—H19C	0.9600
C4—H4A	0.9300	C20—C26	1.371 (3)
C5—C9	1.378 (3)	C20—C21	1.381 (4)
C5—H5A	0.9300	C20—H20A	0.9300
C6—N1	1.280 (2)	C21—C22	1.375 (4)
C6—C9	1.449 (3)	C21—H21A	0.9300
C6—C7	1.501 (3)	C22—C23	1.383 (4)
C7—O2	1.212 (2)	C22—H22A	0.9300
C7—N2	1.367 (3)	C23—C27	1.370 (3)
C8—N2	1.402 (2)	C23—H23A	0.9300
C8—C9	1.401 (3)	C24—N5	1.279 (3)
C10—N4	1.447 (3)	C24—C27	1.450 (3)
C10—H10A	0.9600	C24—C25	1.492 (3)
C10—H10B	0.9600	C25—O6	1.223 (3)
C10—H10C	0.9600	C25—N6	1.354 (3)
C11—O4	1.224 (3)	C26—N6	1.402 (3)
C11—N4	1.360 (3)	C26—C27	1.400 (3)
C11—C12	1.487 (3)	N1—O1	1.385 (2)
C12—N3	1.277 (3)	N3—O3	1.372 (3)
C12—C17	1.448 (3)	N5—O5	1.373 (2)
C13—C14	1.387 (4)	O1—H1B	0.8200
C13—C17	1.389 (3)	O3—H3C	0.8200
C13—H13A	0.9300	O5—H5C	0.8200
C14—C15	1.365 (4)	O7W—H7WA	0.9600
C14—H14A	0.9300	O7W—H7WB	0.9600
			
N2—C1—H1C	109.5	C18—C16—H16A	121.5
N2—C1—H1D	109.5	C13—C17—C18	119.1 (2)
H1C—C1—H1D	109.5	C13—C17—C12	134.5 (2)
N2—C1—H1E	109.5	C18—C17—C12	106.35 (18)
H1C—C1—H1E	109.5	C16—C18—C17	122.6 (2)
H1D—C1—H1E	109.5	C16—C18—N4	127.6 (2)
C8—C2—C3	117.9 (2)	C17—C18—N4	109.79 (18)
C8—C2—H2A	121.1	N6—C19—H19A	109.5
C3—C2—H2A	121.1	N6—C19—H19B	109.5
C4—C3—C2	121.4 (2)	H19A—C19—H19B	109.5
C4—C3—H3A	119.3	N6—C19—H19C	109.5
C2—C3—H3A	119.3	H19A—C19—H19C	109.5
C3—C4—C5	120.7 (2)	H19B—C19—H19C	109.5
C3—C4—H4A	119.6	C26—C20—C21	117.7 (2)
C5—C4—H4A	119.6	C26—C20—H20A	121.2
C9—C5—C4	118.7 (2)	C21—C20—H20A	121.2
C9—C5—H5A	120.7	C22—C21—C20	121.2 (3)
C4—C5—H5A	120.7	C22—C21—H21A	119.4
N1—C6—C9	135.00 (19)	C20—C21—H21A	119.4
N1—C6—C7	117.56 (17)	C21—C22—C23	120.9 (3)
C9—C6—C7	107.45 (16)	C21—C22—H22A	119.5
O2—C7—N2	126.5 (2)	C23—C22—H22A	119.5
O2—C7—C6	128.18 (19)	C27—C23—C22	118.7 (2)
N2—C7—C6	105.36 (16)	C27—C23—H23A	120.7
C2—C8—N2	128.31 (19)	C22—C23—H23A	120.7
C2—C8—C9	121.50 (19)	N5—C24—C27	135.82 (19)
N2—C8—C9	110.19 (16)	N5—C24—C25	117.7 (2)
C5—C9—C8	119.85 (17)	C27—C24—C25	106.41 (18)
C5—C9—C6	134.28 (19)	O6—C25—N6	126.0 (2)
C8—C9—C6	105.86 (17)	O6—C25—C24	127.1 (2)
N4—C10—H10A	109.5	N6—C25—C24	106.86 (19)
N4—C10—H10B	109.5	C20—C26—N6	128.3 (2)
H10A—C10—H10B	109.5	C20—C26—C27	121.6 (2)
N4—C10—H10C	109.5	N6—C26—C27	110.07 (19)
H10A—C10—H10C	109.5	C23—C27—C26	119.9 (2)
H10B—C10—H10C	109.5	C23—C27—C24	133.9 (2)
O4—C11—N4	125.8 (2)	C26—C27—C24	106.19 (18)
O4—C11—C12	127.9 (2)	C6—N1—O1	112.81 (16)
N4—C11—C12	106.29 (18)	C7—N2—C8	111.12 (16)
N3—C12—C17	135.8 (2)	C7—N2—C1	123.47 (17)
N3—C12—C11	117.3 (2)	C8—N2—C1	125.22 (17)
C17—C12—C11	106.83 (18)	C12—N3—O3	112.1 (2)
C14—C13—C17	118.1 (2)	C11—N4—C18	110.72 (17)
C14—C13—H13A	121.0	C11—N4—C10	123.89 (18)
C17—C13—H13A	121.0	C18—N4—C10	125.39 (18)
C15—C14—C13	121.3 (2)	C24—N5—O5	111.20 (18)
C15—C14—H14A	119.3	C25—N6—C26	110.46 (17)
C13—C14—H14A	119.3	C25—N6—C19	124.4 (2)
C14—C15—C16	121.9 (2)	C26—N6—C19	125.0 (2)
C14—C15—H15A	119.1	N1—O1—H1B	109.5
C16—C15—H15A	119.1	N3—O3—H3C	109.5
C15—C16—C18	117.0 (2)	N5—O5—H5C	109.5
C15—C16—H16A	121.5	H7WA—O7W—H7WB	108.0

### Hydrogen-bond geometry (Å, °)

**Table:**

*D*—H···*A*	*D*—H	H···*A*	*D*···*A*	*D*—H···*A*
O7W—H7WA···O6^i^	0.96	1.82	2.752 (3)	164
O1—H1B···N1^ii^	0.82	2.11	2.780 (3)	139
O7W—H7WB···O6^iii^	0.96	2.02	2.958 (3)	165
O7W—H7WB···N5^iii^	0.96	2.59	3.181 (3)	120
O3—H3C···O7W	0.82	1.78	2.579 (3)	165
O5—H5C···O4^iv^	0.82	2.05	2.753 (3)	144

Symmetry codes: (i) *x*, *y*+1, *z*; (ii) −*x*+2, −*y*, −*z*+1; (iii) −*x*+1, −*y*+1, −*z*; (iv) .

### Table 2 Cg···Cg π-ring stacking interactions, Cg1,Cg2,Cg4,Cg5,Cg7,Cg8 are the centroids of rings N2/C6-C8, C2-C5,C8/C9, N4.C11-C12/C17-C18, C13-C17, N6/C24-C27, C20-C23/C27-C28 [Symmetry codes: (i) 1+x,y,z; (ii) x, -1+y, z' (iii) x, y, z; (iv) x, 1+y, z; (v) -1+x, y, z ]

**Table:**

*CgI···CgJ*	*Cg···Cg* (Å)	Cg I_Perp (Å)	CgJ_Perp (Å)
Cg1···Cg7^i,v^	3.5935 (19)	-3.4071 (9)	3.4348 (10)
Cg1···Cg8^i,v^	3.843 (2)	-3.4274 (12)	3.4274 (12)
Cg2···Cg4^ii,iv^	3.7366 (19)	3.5203 (9)	-3.5798 (10)
Cg2···Cg5^ii^	3.7181 (19)	3.5255 (9)	-3.5288 (10)
Cg4···Cg7^iii^	3.983 (2)	3.3212(10	-3.4138 (11)
Cg5···Cg2^iv^	3.7182 (19)	-3.5288 (10)	3.5255 (9)
Cg5···Cg7^iii^	3.4456 (19)	3.4415 (10)	-3.4224 (10)
